# Clodronate disodium does not produce measurable effects on bone metabolism in an exercising, juvenile, large animal model

**DOI:** 10.1371/journal.pone.0300360

**Published:** 2024-04-16

**Authors:** Fernando B. Vergara-Hernandez, Brian D. Nielsen, John M. Popovich, Char L. Panek, Alyssa A. Logan, Cara I. Robison, Richard A. Ehrhardt, Tyler N. Johnson, Nicholas J. Chargo, Thomas H. Welsh, Amanda N. Bradbery, Jessica L. Leatherwood, Aimee C. Colbath

**Affiliations:** 1 Department of Animal Science, College of Agricultural and Natural Resources, Michigan State University, East Lansing, Michigan, United States of America; 2 School of Veterinary Medicine, College of Natural Resources and Veterinary Medicine, Universidad Santo Tomas, Viña del Mar, Chile; 3 Center for Neuromusculoskeletal Clinical Research, College of Osteopathic Medicine, Michigan State University, East Lansing, Michigan, United States of America; 4 Department of Clinical Sciences, College of Veterinary Medicine, Cornell University, Ithaca, New York, United States of America; 5 School of Agriculture, College of Basic and Applied Sciences, Middle Tennessee State University, Murfreesboro, Tennessee, United States of America; 6 Department of Chemical Engineering and Materials Science, College of Engineering, Michigan State University, East Lansing, Michigan, United States of America; 7 Department of Physiology, College of Natural Science, Michigan State University, East Lansing, Michigan, United States of America; 8 Department of Animal Science, College of Agriculture & Life Sciences, Texas A&M University, College Station, Texas, United States of America; 9 Department of Animal and Range Sciences, College of Agriculture, Montana State University, Bozeman, Montana, United States of America; 10 Department of Animal Science, College of Agriculture and Natural Resources, Tarleton State University, Stephenville, Texas, United States of America; University of Life Sciences in Lublin, POLAND

## Abstract

Bisphosphonates are commonly used to treat and prevent bone loss, but their effects in active, juvenile populations are unknown. This study examined the effects of intramuscular clodronate disodium (CLO) on bone turnover, serum bone biomarkers (SBB), bone mineral density (BMD), bone microstructure, biomechanical testing (BT), and cartilage glycosaminoglycan content (GAG) over 165 days. Forty juvenile sheep (253 ± 6 days of age) were divided into four groups: Control (saline), T_0_ (0.6 mg/kg CLO on day 0), T_84_ (0.6 mg/kg CLO on day 84), and T_0+84_ (0.6 mg/kg CLO on days 0 and 84). Sheep were exercised 4 days/week and underwent physical and lameness examinations every 14 days. Blood samples were collected for SBB every 28 days. Microstructure and BMD were calculated from tuber coxae (TC) biopsies (days 84 and 165) and bone healing was assessed by examining the prior biopsy site. BT and GAG were evaluated postmortem. Data, except lameness data, were analyzed using a mixed-effects model; lameness data were analyzed as ordinal data using a cumulative logistic model. CLO did not have any measurable effects on the skeleton of sheep. SBB showed changes over time (p ≤ 0.03), with increases in bone formation and decreases in some bone resorption markers. TC biopsies showed increasing bone volume fraction, trabecular spacing and thickness, and reduced trabecular number on day 165 versus day 84 (p ≤ 0.04). These changes may be attributed to exercise or growth. The absence of a treatment effect may be explained by the lower CLO dose used in large animals compared to humans. Further research is needed to examine whether low doses of bisphosphonates may be used in active juvenile populations for analgesia without evidence of bone changes.

## Introduction

Bisphosphonates are a class of drugs that have been used for over 40 years in human medicine for their antiresorptive biological effects [1, 2]. Bisphosphonates work by impairing osteoclast-mediated bone resorption [3, 4]. As a result, these drugs have been shown to increase bone mineral density (BMD) [5, 6], reduce serum bone biomarkers (SBB) of bone resorption [6, 7], and change the mechanical properties of bones [8, 9]. Bisphosphonates treat and prevent bone loss in various diseases such as postmenopausal osteoporosis [10, 11], osteogenesis imperfecta [12–14], and Paget’s disease [15, 16]. They are also used in veterinary medicine for different pathologies, such as canine osteosarcoma [17], feline idiopathic hypercalcemia [18], and equine navicular syndrome [19, 20].

The effect of bisphosphonates in young, active humans and animals is poorly understood [21–24]. This knowledge gap is particularly significant given the use of bisphosphonates in juvenile, high-performance animals such as racehorses where bone growth and remodeling are active [24–28]. Growth and adaptation to exercise depend on normal osteoclastic activity [29]. Osteoclasts are active and abundant in the subchondral bone of juvenile animals [30] and humans [31]; thus, the potential impairment of this group of cells may result in adverse side effects including decreased bone strength, decreased bone healing, or altered growth [32, 33]. Serious side effects resulting from bisphosphonate use in adults include osteonecrosis of the jaw defined by the accumulation of necrotic bone in the oral cavity [34, 35] and atypical femur fractures which occur in the absence of major injury or trauma [36], have been replicated in several animal models [37–41], but have not been thoroughly investigated in a young, active population.

Bisphosphonate effects have been assessed in different animal models [37–42] including sheep [25]. In comparison to other large animals such as bull calves, sheep can be more easily conditioned to forced exercise [43] and a relatively uniform population can be chosen from select herds with closed breeding practices [44]. Moreover, sheep have been used as a large animal model for horses [44, 45], making them a particularly applicable model species for the effects of therapeutics on active individuals. Further, sheep are amenable to serial blood sampling and bone biopsies, therefore, serving as an important experimental large animal species. When used in a terminal study, bone mechanical testing and skeletal advanced imaging may be combined with gross dissection and sampling, creating a robust data set.

Given the existing knowledge gap regarding the skeletal effects of bisphosphonates on juvenile populations engaged in exercise, this study aimed to investigate the effects of clodronate disodium (CLO) administration on bone formation and remodeling using juvenile sheep subjected to forced exercise. CLO was selected as it is an FDA-approved bisphosphonate for use in a large animal species [46], has shown clinical efficacy for musculoskeletal disease [46, 47], and the pharmacokinetics have been described in sheep [48]. Outcome measures included physical examinations, lameness evaluations, SBB, advanced imaging methods, biomechanical testing (BT), and sulfated glycosaminoglycan content (GAG) in cartilage. Based on the antiresorptive effects of bisphosphonates, we hypothesized that CLO administration would result in (1) a reduction in bone resorption markers indicating a decrease in bone turnover, (2) an increase in BMD and an increase in force to fracture, (3) a decrease in bone healing evident by a decrease in BMD at the bone sampling site, and (4) an increase in cartilage GAG content.

## Materials and methods

### Animals and management

All animal protocols were approved by the Michigan State University (MSU) Institutional Animal Care and Use Committee (2020000264) and the experimental procedures and results are described according to the Animal Research: Reporting of In Vivo Experiments (ARRIVE) guidelines [49]. Forty juvenile, cross bred Dorset-Polypay sheep (253 ± 6 days of age), consisting of 20 castrated males (wethers, 87 ± 5 kg), and 20 females (ewes, 72 ± 8 kg) were acquired from the MSU Sheep Teaching and Research Center. Dorset-Polypay sheep were selected due to their availability at the MSU Sheep Farm, ensuring a population of a similar age, under consistent management conditions and with both sexes represented. Castrated males were used as the farm orchiectomizes the rams (intact males) shortly after birth. Juvenile sheep were defined as animals that had not attained 80% of their mature size (body mass) [50, 51]. To determine the growth rate of the sheep, the sheep were weighed every two weeks, beginning 150 days prior to the start of the study. At the start of the study, sheep were less than 80% of their projected, mature size. Sheep were sheared nine weeks prior to the beginning of the study. Two weeks prior to the beginning of the study, sheep were transported to the MSU Bennett Road Farm and then housed in two indoor pens (21.6 m^2^ each, S1 Fig). The study was comprised of a 2-week acclimation period followed by a 24-week study period. Animals were randomly assigned to each indoor pen, with 20 animals housed in each pen. Sheep had access to a total mixed ration that contained 90% dry matter of chopped hay (82%), a corn/soybean blend (16.5%), and a 1.5% of a mineral blend (Marvo Mineral Company, Osseo, MI). The sheep were fed this total mixed ration once per day at a mass equivalent to an average of 2.0–2.3% of their body weight in dry matter over the 24-week experimental period and were provided ad libitum access to water.

**Fig 1:**
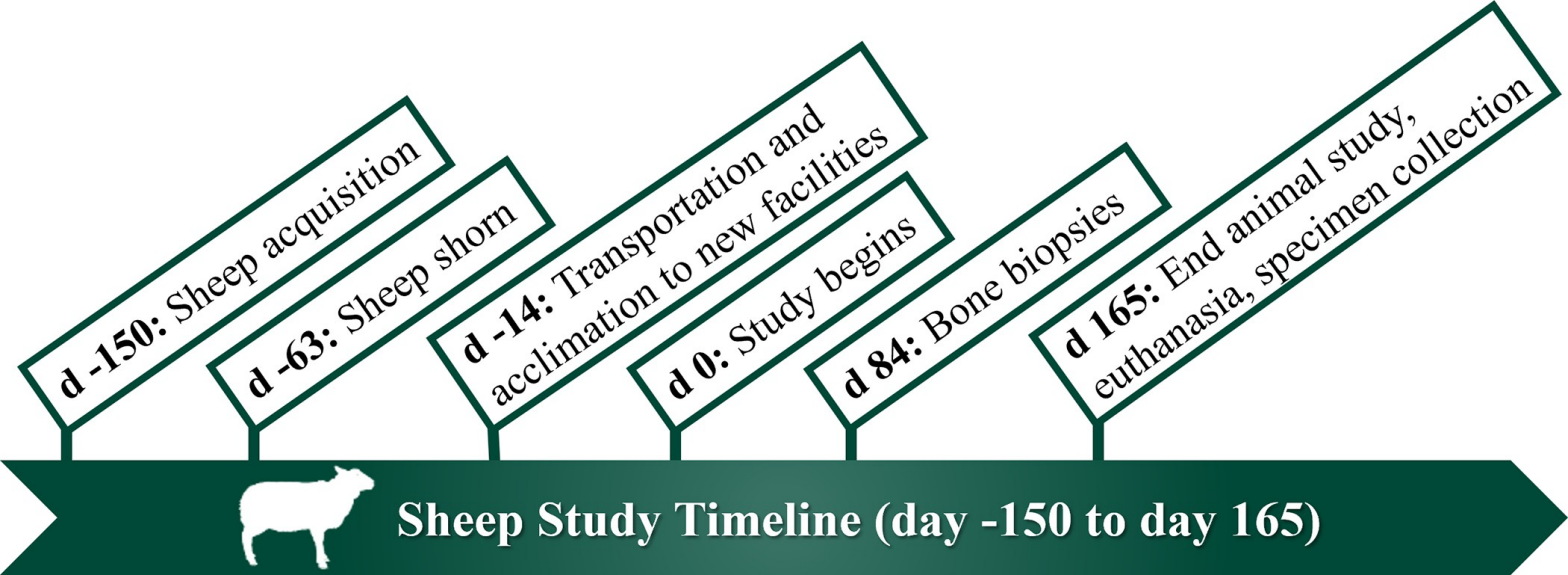
Study timeline. Sheep were acquired at day -150 to allow for early health screening and weighing. Sheep were transported to the exercise facility at day -14 for a 2-week acclimation period prior to the start of the study. The total study period was 165 days, and all sheep underwent a tuber coxae bone biopsy procedure at 84 days.

During the 2-week habituation period, sheep were acclimated to their housing, a 3-way manual weigh crate (Prattley™, Temuka, New Zealand), and the 20-m diameter exerciser (Q-Line Horse Exerciser, Aromas, CA, USA). Twice per week, animals were moved through an indoor chute system connected to the weigh crate to acclimate the sheep to sample collections and physical examinations. A 32-meter fence connecting the outside walker to the inside pens allowed sheep to easily move from the barn to the walker. During the habituation period, sheep were placed in the exerciser 4x/week and encouraged to walk for 5 min/day (S1 Fig). Fig 1 describes the study timeline.

### Physical examinations and exercise protocol

Physical examinations (PE) were performed every 14 days during the study period, and included body weight (BW) measured using a weigh crate, height measured at the withers (height) using a solid measuring stick, heart rate (HR) and respiratory rate (RR) determined by auscultation of the heart and trachea, and rectal temperature (RT) acquired by digital thermometer. All physical examinations were performed using the previously mentioned indoor chute system and weigh crate. Sheep were confirmed to be without lameness prior to the start of the study, and lameness evaluations were performed every 14 days starting on week 0 by two veterinarians (AC, FV) using a standardized sheep subjective lameness evaluation scoring system that ranged from “0” to “6” [52]. According to this scoring system a score of “0” denotes no lameness, a score of “1” is an irregular posture without any reduction in stride length, a score of “2” indicates a noticeable head nod accompanied by a shortened stride, a score of “3” is given to an animal showing significant discomfort while moving, as evidenced by excessive head flicking and stride shortening, a score of “4” is given when the animal shows a reluctance to bear weight during movement, a score of “5” is assigned when the animal is unable to stand up and shows a reluctance to move, and a score of “6” when the animal is unable to stand or move [52].

An exercise protocol was developed for the sheep, with its intensity and duration based on prior treadmill sheep studies [45, 53]. This protocol was designed to emulate exercise protocols used in juvenile horse studies [54, 55]. Supervised by a veterinarian (FV), the exercise sessions took place between 07:00 and 10:00 h. The sheep started with a brisk 10-minute walk each day with the duration extended by 5 minutes weekly until it reached a daily maximum of 30 minutes. At this point, a strenuous pace for the sheep (2.0 m/s) was incorporated into the middle of the workout [53]. The sheep underwent exercise four times a week, with the direction of exercise alternating between clockwise and counterclockwise each day. The sheep were exercised in groups of 20 to ensure their safety, as the exerciser’s bay could not accommodate all 40 sheep at once, and to maintain the original distribution of animals from each pen. No exercise was completed during week 12 as sheep were sedated and bone biopsies were collected from the tuber coxae (TC); sheep resumed exercise during week 13 (Table 1).

**Table 1:** Juvenile sheep exercise protocol and estimated distance traveled using a circular exerciser for 24 weeks.

Week	Exercise duration	Estimated distance traveled
1	10 min (WS)	780 m
2	15 min (WS)	1,170 m
3	20 min (WS)	1,560 m
4	25 min (WS)	1,950 m
5	30 min (WS)	2,340 m
6–8	13 ¾ min (WS) → 2 ½ min (TS) → 13 ¾ min (WS)	2,445 m
9–11	12 ½ min (WS) → 5 min (TS) → 12 ½ min (WS)	2,550 m
12^1^	–	–
13	30 min (WS)	2,340 m
14	12 ½ min (WS) → 5 min (TS) → 12 ½ min (WS)	2,550 m
15–17	8 min (WS) → 3 ¾ min (TS) → 6 ½ min (WS) → 3 ¾ min (TS) → 8 min (WS)	2,655 m
18–24	7 min (WS) → 5 min (TS) → 6 min (WS) → 5 min (TS) → 7 min (WS)	2,760 m

Abbreviations: WS, walking speed at 1.3 m/s; TS, trotting speed at 2.0 m/s. Sheep were exercised 4x/week alternating between clockwise and counterclockwise directions each day. The TS bout was incorporated in the middle of the 30 min exercise protocol, starting on week 6.

^1^No exercise due to bone biopsies.

### Treatment distribution

An a priori power analysis, with a significance criterion of alpha = 0.05, was conducted based on mean differences and pooled standard deviations (SD) between treatment groups for bone-specific alkaline phosphatase (BALP, bone formation marker) and cross-linked C-terminal telopeptides of type I collagen (CTX-I, bone resorption marker). The analysis revealed that a sample size of 10 animals per treatment group would provide sufficient power to detect a significant difference. For BALP, the estimated power was 99.9% with a mean difference of 7 ng/mL and a SD of 3 ng/mL [56]. For CTX-I, the estimated power was 98.1% with a mean difference of 135 ng/mL and a SD of 75 ng/mL using an ELISA method [38]. Sample size and power were calculated using OpenEpi (Version 3.01). The authors recognized the limited information available on bisphosphonate administration in juvenile sheep and, therefore, maximized the power of the study by using 10 animals per treatment group.

Sheep were stratified by sex and weight, randomly assigned a number from #1 to #40, and allocated to one of three CLO treatment groups (T_0,_ T_84,_ T_0+84_) or a saline control group (Con), with each group consisting of 10 sheep. Treatment groups received CLO (0.6 mg/kg (0.01ml/kg),OSPHOS^®^, Dechra Veterinary Products, Overland Park, KS, USA) administered on day 0 (T_0_), CLO (0.6 mg/kg) administered on day 84 (T_84_), or CLO (0.6 mg/kg) administered on day 0 and day 84 (T_0+84_). All doses were administered intramuscularly (i.m.). To maintain proper controls, animals not receiving CLO on days 0 or 84 was administered saline solution i.m., and control animals received saline solution i.m. on days 0 and 84. The dose of CLO was selected based on a pilot study which compared plasma concentrations of CLO following 3 different doses of CLO (0.6, 1.8, 3.0 mg/kg i.m.) over 48 hours in 12 adult sheep (*n* = 4/treatment group) [48]. CLO administered at 0.6 mg/kg i.m. resulted in similar pharmacokinetic parameters to those observed with a dose of CLO at1.8 mg/kg i.m. for horses [48].

### Serum harvest

Twenty milliliters of blood were harvested by jugular venipuncture between 07:00 and 09:00 h every 28 days, starting on day 0. Blood was placed in serum-separator vacutainer tubes, facilitating coagulation on ice for 1 h prior to centrifugation at 2,000 × *g* for 15 min. Serum was aliquoted into 2-mL microcentrifuge tubes and stored at -80°C for later evaluation.

### Tuber coxae biopsy

At week 12, TC bone biopsies were performed to assess bone healing and microstructure. To ensure unbiased sampling and facilitate blinded post-mortem analysis, the left or right TC was randomly chosen regardless of the treatment group, using a random sequence generator (https://www.random.org/sequences). Sheep were fasted for 24 h and deprived of water for 12 h prior to surgery. Sheep received a single dose of penicillin G procaine (22,000 UI/kg i.m.; VetOne, Boise, ID, USA) prior to administration of midazolam (0.26 mg/kg i.m.; Hikma Pharmaceuticals USA Inc., Berkeley Heights, NJ, USA), ketamine (3.25 mg/kg i.m.; Akorn Operating Company LLC, Gurnee, IL, USA), and xylazine (0.01–0.02 mg/kg i.m. as needed; Patterson Veterinary Supply Inc., Loveland, CO, USA) to achieve recumbent sedation. The biopsy site was clipped and aseptically prepared, and the skin was infiltrated with 5 mL of mepivacaine hydrochloride. An approximately 4-cm incision was made through the skin and subcutaneous tissues over the TC. An 8-mm Michele trephine was used to remove a sample which included a cartilage cap, cortical bone, and trabecular bone. Biopsy samples were stored in 4% paraformaldehyde solution for 96 h and then transferred to 70% ethanol for later micro-computed tomography (micro-CT) analysis. The subcutaneous tissue was closed using a 0-monocryl suture in a continuous pattern and the skin incision was closed using a 2-0-monocryl suture in a simple, interrupted pattern. All animals were administered a single dose of meloxicam (1 mg/kg PO; Zydus Pharmaceuticals Inc., Pennington, NJ, USA) after the skin incision was sutured.

### Euthanasia and specimen collection

At the conclusion of the 24-week study, all sheep were humanely euthanized using a captive bolt pistol at the MSU Meat Laboratory. This method was chosen for its efficiency and to minimize chemical contamination of tissues. Mandibles, right fused metacarpi (MC_3+4_), and fourth lumbar vertebrae (L4) were collected from each animal, wrapped in saline-soaked paper towels, stored in plastic bags, and immediately placed on ice. The medial condyle of the left MC_3+4_ (LMC) and the third lumbar vertebra (L3) were placed in 4% paraformaldehyde. The TC which had been previously biopsied (during week 12) was removed en bloc and an 8-mm biopsy was obtained from the contralateral TC for preservation in 4% paraformaldehyde. All samples in 4% paraformaldehyde were transferred to 70% ethanol after 96 h. Articular cartilage was harvested from the proximal surface of the right radius using a scalpel. The harvested cartilage was immediately placed in microcentrifuge tubes and stored on ice, then transferred to storage at -20°C until GAG analysis.

### Computed tomography

The mandible, right MC_3+4_, and L4 from each sheep were CT scanned at 120 kV and 320 mAmp, with a slice thickness of 0.625 mm (GE Revolution Evo Scanner; GE Healthcare, Princeton, NJ, USA). All CT scans were analyzed using Mimics 23.0 (Materialise NV, Leuven, Belgium). The whole-slice BMD of the mandible was calculated with a mask threshold of 250 Hounsfield Units (HU) at the midpoint of the diastema between the fourth incisor and premolar; the BMD of the left and right hemi-mandibles was averaged. The midpoint of each right MC_3+4_ was used to measure BMD and the following dimensions with a mask threshold value of 400 HU: cross-sectional area (CSA), external dorsopalmar (DP) and lateromedial (LM) diameters (cortex), internal DP and LM diameters (medullary cavity), and cortical widths (anterior, posterior, lateral, and medial). The BMD of L4 was measured at the first full cranial slice, midpoint, and last full caudal slice and averaged. Additionally, the vertebral body length and CSA dimensions of the L4 were measured. To calculate the CSA, the average cranial and caudal full slice areas were used. Vertebral scans were identified and separated by masking at 226 HU. A calcium hydroxyapatite phantom (Image Analysis, Inc., Columbia, KY, USA) with rows representing 0, 75, and 150 milligrams of calcium hydroxyapatite per cubic centimeter (mg HA/cm^3^) was included in each scan. All density measurements in HU were converted to mg HA/cm^3^ using linear equations calculated from the phantom on each scan, as previously described [57]. After CT scans were completed, all samples were wrapped in saline-soaked gauze and stored at -20°C until later analysis by BT.

### Biomechanical testing

The right MC_3+4_ were removed from the freezer, wrapped in saline-soaked gauze, and allowed to slow thaw at 4.8°C over a 5-day period. Soft tissue was removed after thawing and immediately prior to biomechanical testing. The right MC_3+4_ were subjected to 4-point bending using an electromechanical testing system equipped with a 60 kN load cell (MTSCriterion, Model 43, Eden Prairie, MN, USA). Right MC_3+4_ for each sheep were positioned individually, with the palmar aspect of the metacarpus facing upward toward the force applicators, as previously described [43] (S2 Fig). All samples were loaded to failure at a rate of 10 mm/min [58]. Flexural stress (maximal force to failure/CSA) and modulus of elasticity were calculated. Modulus of elasticity (*E*) was calculated based on the formula: *E = EI/I*. The *EI* (flexural rigidity) was calculated by the following equation: $EI=(F/V)(a^{2}/12)(3\times L–4a)$, where *F/V* (stiffness) was obtained from the linear portion of the curve between 0.7 to 1.2 mm of compression with an R^2^ of 0.99. The distance between the bottom support stands minus the width of the support stands was *L* (51.9 mm); *a* (3.8 mm) corresponds to *L* minus the distance between the force applicators divided by two (S2 Fig). Moment of inertia (*I*) was determined by calculating a hollow ellipse as previously described [43, 59]: $I=0.049[(B\times D^{3})–(b\times d^{3})]$ (*B* = exterior lateromedial diameter, *D* = exterior dorsopalmar diameter, *b* = interior lateromedial diameter, *d* = interior dorsopalmar diameter).

L4 were allowed to thaw at 4.8°C for 3 days prior to the removal of soft tissues. Once thawed, the soft tissues, vertebral arch, and transverse processes were removed (S3 Fig). The superior and inferior vertebral endplates were embedded in polyurethane resin (TC-808, BJB Enterprises, Tustin, CA, USA) (S4 Fig) and the specimens were wrapped in saline-soaked gauze. Compression tests were performed using an electromechanical testing system equipped with a 100 kN load cell (Instron Model 5982, Norwood, MA, USA). A flat 3-mm thick metal plate was included between the specimen and the compression piston’s end to guarantee a uniformly distributed axial load applied over the vertebral body (S5 Fig). All specimens were loaded to failure at a rate of 1 mm/min [58]. Compressive stress (maximal compressive force divided by the average CSA calculated from the CT data for each vertebral body) was calculated. Compressive modulus of elasticity was calculated as the slope of the stress-strain curve between 60 to 80% of the maximum compressive stress using the Bluehill^®^ Universal software (Norwood, MA, USA).

### Micro-computed tomographic analysis

Fixed whole TC, TC biopsies, LMC, and L3 vertebral bodies were analyzed using micro-computed tomography (micro-CT). Micro-CT images were obtained using a PerkinElmer Quantum GX system (Waltham, MA, USA) with a voltage of 90 kV, current of 88 μA, and reconstruction resolution of 50 μm. Image analysis was performed using Dragonfly Software (v.2022.1.0.1259, Object Research Systems, Quebec, Canada) to differentiate bone (mineral) from bone marrow (non-mineral) and segment TC biopsy slices using the Otsu threshold algorithm [60]. The Otsu method performs clustering-based image thresholding that is determined by minimizing intra-class intensity variance, or by maximizing inter-class variance. [60] This method has been used previously to segment bone tissue [61–66] and has been suggested to be used for objective analysis of micro-CT studies [67], due to the objective nature of the algorithm. Thus, the Otsu method was chosen for its ability to provide an unbiased segmentation.

For TC biopsies, a cylindrical region of interest (ROI) was established for the calculation of bone volume fraction (BV/TV), trabecular separation (TbSp), trabecular thickness (TbTh), trabecular number (TbN), trabecular connectivity density (ConnD), and BMD. To assess bone healing, a 6-mm diameter cylinder ROI was established using the Otsu threshold tool and the Bone Analysis tool was used to calculate BV/TV, TbSp, TbTh, TbN, and ConnD at the TC biopsy site. Cortical thickness (CtTh) was determined by measuring and averaging the cortical bone thickness in 5 different regions of a representative slice of the TC. L3 and LMC were segmented using the Otsu threshold tool. Separation of cortical from the trabecular bone in L3 and LMC was done using the Buie bone segmentation tool in the Bone Analysis tool [67], resulting in the calculation of BV/TV, TbSp, TbTh, TbN, CtTh, and BMD. Growth plates were present in the LMC and L3 samples; therefore, those regions were excluded from the ROI to standardize image analysis. [57] During each micro-CT imaging session, a micro-CT calcium hydroxyapatite phantom (QRM, Möhrendorf, Germany) that consisted of five cylindrical inserts containing various densities of calcium hydroxyapatite (0, 50, 200, 800 and 1200 mg HA/cm^3^) was scanned and used to create a linear calibration between Hounsfield units and these known phantom densities. For the micro-CT images, BMD was calculated using the average bone Hounsfield units and converted to mg HA/cm^3^ using the linear calibration, as previously described [57].”

### Serum bone biomarker analysis

Serum samples were thawed immediately before testing. Serum concentrations of ovine-specific bone markers [68] were assessed using enzyme-linked immunoassays according to the manufacturer’s instructions (Kendall Scientific, Lincolnshire, IL, USA). Bone markers analyzed included BALP, procollagen type I amino-terminal propeptide (PINP), receptor activator of nuclear factor NF-ĸB ligand (RANKL), CTX-I, and tartrate-resistant acid phosphatase isoenzyme 5b (TRAP5b). The assays were analyzed using a SpectraMax ABS Microplate Reader (Molecular Devices, LLC, San Jose, CA, USA) at 450 nm. The remodeling index BALP/CTX-I [69, 70] and resorption index CTX-I/TRAP5b [71, 72] were also determined.

### Cartilage glycosaminoglycan content

Cartilage samples were thawed, weighed, digested in papain buffer (0.1M sodium acetate, 0.05 EDTA, pH 5.53), and activated with 0.005M L-cysteine HCl hydrate overnight in a 60°C water bath. One μg of papain (26 mg/mL) was added per milligram of cartilage, as previously described [73]. The digested GAG content was determined using a 1,9-dimethylmethylene blue colorimetric assay as previously described [74]. The colorimetric reaction was measured at 520 nm using a SpectraMax 384 Microplate Reader (Molecular Devices) and compared to a chondroitin sulfate standard (bovine trachea).

### Statistical analysis

Physical parameters and SBB of sheep were analyzed using a mixed-effects model that considered the fixed effects of treatment, time, sex, and all possible 2- and 3-way interactions, with repeated measures of time and subject effect of sheep. The results are presented as mean values ± SD and were analyzed using the MIXED procedure of SAS 9.4 (SAS Inc., Cary, NC, USA). CT (mandibles, right MC_3+4_, and L4), micro-CT (TC, TC biopsies, L3, and LMC), BT data (right MC_3+4_ and L4), and cartilage GAG content were evaluated with the fixed effects of treatment, sex, as well as the interaction between treatment and sex. Normality was assessed using diagnostic plots of residuals for each independent variable. All data, except for SBB and lameness evaluations, were deemed to be normally distributed. SBB data was log-transformed and subsequently followed a normal distribution after transformation. Height and BW were assessed as potential covariates in this model, because the size of the animals could have influenced the results of the independent variables. No significant correlations were detected between height or BW and the independent variables; therefore, they were removed from the final model. An effect of sex was observed in sheep BW, height, right MC_3+4_ dimensions, L4 dimensions and BT, and LMC BV/TV and BMD. For the rest of the analyses, the effect of sex was not included in the results, as it was not significant. Post-hoc comparisons using least-squares means separated by the Tukey-Kramer test were used when the effects were significant (p ≤ 0.05).

Lameness data were analyzed as ordinal data. Lameness scores from two veterinarians were averaged and rounded to the nearest integer for each sheep. A cumulative logistic model was applied using RStudio v.2022.07.2 (RStudio Inc., Boston, MA, USA), calculating the estimated likelihood of lameness score, measured as predicted probability for each treatment and day and standard error. Post-hoc multiple comparisons were performed using the Tukey method and the R package ’emmeans’. Statistical significance was set at p ≤ 0.05.

## Results

### Physical examinations and lameness evaluations results

During the study, two sheep experienced moderate morbidity leading to intervention. During week 8, sheep #7 (female, T_0_ group) developed a fever and lethargy; she was treated with penicillin G procaine (22,000 UI/kg i.m.) for 10 days every 12 h (7 days of exercise missed). She fully recovered until week 12, when she experienced persistent lameness following TC biopsy. Due to persistent lameness, exercise was discontinued, and sheep #7 was excluded from the study. One additional sheep developed a mild fever and lethargy following TC biopsy (#37, female, T_84_ group) and received oxytetracycline (20 mg/kg) every 48 h, receiving 2 doses in total (no days of exercise missed).

As anticipated with growth, BW and height increased over time (P < 0.001) but did not differ with treatment administration over time (P ≥ 0.24, Table 2).

**Table 2:** Initial and final body weights (BW) and heights of 40 juvenile sheep.

**Sex**	**BW (d 0)**	**BW (d 163)**	**P-value**
Males (kg)	79 ± 7^b^	86 ± 6^a^	0.01
Females (kg)	70 ± 7^c^	74 ± 9^bc^	
Average BW (kg)	75 ± 8	80 ± 10	< 0.001
**Sex**	**Height (d 0)**	**Height (d 163)**	**P-value**
Males (cm)	69 ± 3^x^	80 ± 2^z^	0.01
Females (cm)	67 ± 3^x^	75 ± 4^y^	
Average height (cm)	68 ± 3	77 ± 4	< 0.001

^abc^BW means followed by a common letter are not significantly different (p ≤ 0.01)

^xyz^Height means followed by a common letter are not significantly different (p ≤ 0.01)

Values are expressed as mean ± SD.

### Lameness evaluations

There were no differences in lameness between treatment groups at any time point. However, on day 94, sheep in the T_0_ group were less likely to be sound (score “0”, 0.53 ± 0.10, p ≤ 0.05) compared to days 0, 30, 44, 58, 72, 114, 142 and 163. Additionally, they were more likely to receive a score of “1” (0.21 ± 0.04, p ≤ 0.05) compared to days 30, 72, 114, and 142. A single sheep from the T_0_ group (sheep #7) had a lameness score of “4” (reluctance to bear weight during movement) at day 94 following TC biopsy. This lameness persisted despite treatment with meloxicam (1 mg/kg PO) every 24 h for five days. Because the animal could not complete the exercise protocol, it was removed from further analyses, as previously indicated. No other sheep received a score greater than “2” at any point during the study period. Table 3 shows the frequency for each lameness score according to their treatment groups and days.

**Table 3:** Frequency of sheep lameness scores by day and treatment group.

**Con**	**Day**	**Frequency of lameness score**					**T**_**0**_	**Day**	**Frequency of lameness score**				
		**0**	**1**	**2**	**3**	**4**			**0**	**1**	**2**	**3**	**4**
	0	10	0	0	0	0		0	8	2	0	0	0
	16	9	0	1	0	0		16	9	0	1	0	0
	30	8	1	1	0	0		30	9	1	0	0	0
	44	8	2	0	0	0		44	10	0	0	0	0
	58	10	0	0	0	0		58	10	0	0	0	0
	72	10	0	0	0	0		72	10	0	0	0	0
	94^1^	5	3	2	0	0		94^1^	6	3	0	0	1
	100	8	2	0	0	0		100	9	0	0	1	0
	114	10	0	0	0	0		114	9	0	1	0	0
	128	9	1	0	0	0		128	7	0	3	0	0
	142	10	0	0	0	0		142	9	0	1	0	0
	156	9	0	1	0	0		156	9	0	1	0	0
	163	10	0	0	0	0		163	9	0	1	0	0
	Total observations	116	9	5	0	0		Total observations	114	6	8	1	1
**T**_**84**_	**Day**	**Frequency of lameness score**					**T**_**0+84**_	**Day**	**Frequency of lameness score**				
		**0**	**1**	**2**	**3**	**4**			**0**	**1**	**2**	**3**	**4**
	0	10	0	0	0	0		0	10	0	0	0	0
	16	10	0	0	0	0		16	9	0	1	0	0
	30	10	0	0	0	0		30	10	0	0	0	0
	44	10	0	0	0	0		44	9	1	0	0	0
	58	10	0	0	0	0		58	8	1	1	0	0
	72	9	0	1	0	0		72	10	0	0	0	0
	94^1^	7	2	1	0	0		94^1^	8	2	0	0	0
	100	9	0	1	0	0		100	10	0	0	0	0
	114	9	1	0	0	0		114	10	0	0	0	0
	128	9	0	1	0	0		128	10	0	0	0	0
	142	10	0	0	0	0		142	10	0	0	0	0
	156	10	0	0	0	0		156	9	1	0	0	0
	163	10	0	0	0	0		163	9	1	0	0	0
	Total observations	123	3	4	0	0		Total observations	122	6	2	0	0

Abbreviations: Con, control group with no drugs administered; T_0_, group treated once on day 0 with clodronate disodium (CLO); T_84_, group treated once on day 84 with CLO; T_0+84_ group treated on day 0 and 84 with CLO.

No lameness evaluations were recorded between 5–6.

^1^Lameness evaluations were recorded on day 94 instead of day 86 due to bone biopsies and lack of exercise during week 12.

### Computed tomography

No treatment differences or sex differences were found in the BMD of the mandibular, MC_3+4_ cortices and whole slice at the midpoint, or averaged L4 (Table 4).

**Table 4:** Treatment means and SD of bone mineral density (BMD) measured using computed tomography at various locations: Midpoint of the mandibular diastema, dorsal, palmar, lateral, medial cortices, whole slice at the midpoint of the right fused metacarpus (MC_3+4_), and average of the fourth lumbar vertebral body (L4).

	BMD (mg HA/cm^3^)						
Treatments	Mandible	MC_3+4_					L4
	Midpoint diastema	Dorsal cortex	Palmar cortex	Lateral cortex	Medial cortex	Midpoint whole slice	Vertebral body
Con	805 ± 74	1,180 ± 85	767 ± 145	1,201 ± 67	1,179 ± 60	901 ± 140	390 ± 35
T_0_	813 ± 65	1,209 ± 69	765 ± 143	1,213 ± 35	1,240 ± 55	898 ± 143	426 ± 29
T_84_	809 ± 51	1,203 ± 61	717 ± 93	1,207 ± 35	1,233 ± 52	834 ± 87	421 ± 50
T_0+84_	808 ± 53	1,202 ± 85	792 ± 153	1,193 ± 64	1,225 ± 90	963 ± 161	403 ± 45
P-value	0.99	0.82	0.68	0.88	0.21	0.24	0.22

Abbreviations: Con, control treatment no drugs administered; T_0_, group treated once on day 0 with clodronate disodium (CLO); T_84_, group treated once on day 84 with CLO; T_0+84_ group treated on day 0 and 84 with CLO.

No treatment differences were found for any of the right MC_3+4_ dimensions. However, sex differences were found in all right MC_3+4_ dimensions. Males had greater external and internal DP and LM diameters (p ≤ 0.03), as well as dorsal, palmar, lateral, medial CW, CSA, and bone length (p ≤ 0.02). The L4 vertebral body dimensions (length and CSA) did not differ among treatments, but males had greater vertebral body length and CSA measurements than females (P<0.003, Table 5).

**Table 5:** Means and SD for the right fused metacarpus (MC_3+4_) including the internal and external diameters at the dorsopalmar (DP) and lateromedial (LM) locations, cortical widths (CW) at the dorsal, palmar, lateral, and medial cortex, bone length, cross-sectional area (CSA) and means and SD for the fourth lumbar vertebral body (L4) CSA.

**Measurements**	**MC**_**3+4**_ **dimensions**				
	**Con**	**T**_**0**_	**T**_**84**_	**T**_**0+84**_	**P-value**
DP external diameter (mm)	13.6 ± 0.9	13.9 ± 0.7	13.5 ± 1.0	13.6 ± 0.8	0.31
DP internal diameter (mm)	7.4 ± 0.8	7.7 ± 0.8	7.0 ± 0.6	7.3 ± 0.9	0.19
LM external diameter (mm)	20.0 ± 1.0	20.9 ± 1.5	20.2 ± 1.3	20.6 ± 1.4	0.26
LM internal diameter (mm)	12.0 ± 1.0	12.6 ± 1.7	11.6 ± 1.1	12.2 ± 1.5	0.34
Dorsal CW (mm)	3.8 ± 0.5	4.0 ± 0.5	4.0 ± 0.5	3.9 ± 0.5	0.79
Palmar CW (mm)	2.4 ± 0.3	2.4 ± 0.2	2.5 ± 0.3	2.4 ± 0.2	0.81
Lateral CW (mm)	4.1 ± 0.4	4.1 ± 0.4	4.2 ± 0.3	4.2 ± 0.3	0.67
Medial CW (mm)	4.0 ± 0.4	4.2 ± 0.4	4.4 ± 0.4	4.2 ± 0.4	0.11
Bone length (mm)	150.8 ± 9.7	154.0 ± 9.1	150.6 ± 9.9	150.6 ± 8.2	0.69
CSA (mm^2^)	214.5 ± 25.3	235.9 ± 30.0	216.3 ± 31.4	221.8 ± 30.9	0.25
	**Males**		**Females**		**P-value**
DP external diameter (mm)	14.2 ± 0.6		13.1 ± 0.7		< 0.001
DP internal diameter (mm)	7.7 ± 0.8		7.0 ± 0.8		0.02
LM external diameter (mm)	21.3 ± 1.0		19.6 ± 1.1		< 0.001
LM internal diameter (mm)	12.6 ± 1.4		11.6 ± 1.2		0.03
Dorsal CW (mm)	4.1 ± 0.4		3.7 ± 0.4		0.003
Palmar CW (mm)	2.5 ± 0.3		2.3 ± 0.2		0.03
Lateral CW (mm)	4.3 ± 0.2		4.0 ± 0.3		0.003
Medial CW (mm)	4.4 ± 0.4		4.1 ± 0.4		0.02
Bone length (mm)	158.8 ± 4.7		143.5 ± 4.6		< 0.001
CSA (mm^2^)	240.2 ± 21.8		202.3 ± 23.6		< 0.001
**Measurements**	**L4 dimensions**				
	**Con**	**T**_**0**_	**T**_**84**_	**T**_**0+84**_	**P-value**
Vertebral body length (mm)	39.1 ± 1.9	39.2 ± 1.9	39.1 ± 1.6	39.0 ± 2.2	0.69
CSA (mm^2^)	561 ± 48	593 ± 80	563 ± 48	582 ± 73	0.25
	**Males**		**Females**		**P-value**
Vertebral body length (mm)	40.1 ± 1.8		38.3 ± 1.5		0.003
CSA (mm^2^)	619 ± 50		540 ± 44		< 0.001

Abbreviations: Con, control treatment no drugs administered; T_0_, group treated once on day 0 with clodronate disodium (CLO); T_84_, group treated once on day 84 with CLO; T_0+84_ group treated on day 0 and 84 with CLO.

### Biomechanical testing

No treatment, sex, or treatment-by-sex interaction differences were found in any BT values for the right MC_3+4_. No treatment differences were found for L4 BT variables. However, sex differences were observed. Females had greater compressive stress (P<0.001) and males had a greater modulus of elasticity (p = 0.04, Table 6).

**Table 6:** Means and SD for flexural stress (N/mm^2^) and modulus of elasticity (GPa) reported for the right fused metacarpus (MC_3+4_). Compressive stress and modulus of elasticity are reported for the fourth lumbar vertebra (L4).

**Treatments**	**MC**_**3+4**_		**L4**	
	**Flexural stress (N/mm** ^**2**^ **)**	**Modulus of elasticity** **(GPa)**	**Compressive stress (N/mm** ^**2**^ **)**	**Modulus of elasticity** **(GPa)**
Con	17.9 ± 4.0	3.0 ± 0.7	22.4 ± 3.5	25.2 ± 5.5
T_0_	17.2 ± 5.9	3.1 ± 0.7	21.6 ± 5.1	30.7 ± 4.3
T_84_	19.3 ± 4.9	3.3 ± 0.7	23.8 ± 3.4	29.5 ± 5.1
T_0+84_	18.2 ± 4.7	3.2 ± 0.7	21.1 ± 3.8	26.9 ± 6.7
P-value	0.84	0.77	0.30	0.13
**Sex**	**Flexural stress (N/mm** ^**2**^ **)**	**Modulus of elasticity** **(GPa)**	**Compressive stress (N/mm** ^**2**^ **)**	**Modulus of elasticity (GPa)**
Males	18.7 ± 5.5	2.9 ± 0.5	19.9 ± 3.3	29.8 ± 6.4
Females	17.6 ± 4.0	3.4 ± 0.8	24.8 ± 3.0	26.1 ± 4.3
P-value	0.49	0.09	< 0.001	0.04

Abbreviations: Con, control treatment no drugs administered; T_0_, group treated once on day 0 with clodronate disodium (CLO); T_84_, group treated once on day 84 with CLO; T_0+84_ group treated on day 0 and 84 with CLO; GPa, Gigapascals.

### Micro-computed tomography

No treatment differences were found for the BV/TV, TbSp, TbTh, TbN, ConnD, and BMD of the biopsy site and CtTh of the whole TC (Table 7). Similarly, no treatment-by-time differences were found for BV/TV, TbSp, TbTh, TbN, ConnD, and BMD of TC biopsies. However, time differences were observed for some of the TC biopsy parameters. On day 165, BV/TV, TbSp, and TbTh were increased compared to day 84 (p ≤ 0.04), and TbN was decreased compared to day 84 (P<0.001). No time differences were found for ConnD or BMD (Table 8).

**Table 7:** Treatment means and SD of the bone healing measured as bone volume fraction (BV/TV), trabecular separation (TbSp), trabecular thickness (TbTh), trabecular number (TbN), trabecular connectivity density (ConnD), and bone mineral density (BMD) of the tuber coxae (TC) biopsy site and whole TC cortical thickness (CtTh).

Measurement	TC Bone healing				
	Con	T_0_	T_84_	T_0+84_	P-Value
BV/TV (%)	33.6 ± 6.3	32.6 ± 7.2	38.3 ± 11.0	33.7 ± 9.4	0.47
TbSp (mm)	1.87 ± 0.64	1.76 ± 0.85	1.64 ± 0.82	1.56 ± 0.73	0.81
TbTh (mm)	0.58 ± 0.10	0.53 ± 0.10	0.59 ± 0.08	0.50 ± 0.12	0.18
TbN (mm^-1^)	0.44 ± 0.11	0.52 ± 0.26	0.50 ± 0.15	0.55 ± 0.21	0.59
ConnD (mm^-3^)	6.7 ± 4.6	7.0 ± 3.3	6.0 ± 3.3	6.9 ± 3.9	0.95
BMD (mg HA/cm^3^)	238 ± 60	277 ± 54	292 ± 63	281 ± 80	0.29
TC CtTh (mm)	0.86 ± 0.10	0.86 ± 0.11	0.85 ± 0.13	0.84 ± 0.05	0.98

Abbreviations: Con, control treatment no drugs administered; T_0_, group treated once on day 0 with clodronate disodium (CLO); T_84_, group treated once on day 84 with CLO; T_0+84_ group treated on day 0 and 84 with CLO.

**Table 8:** Treatment means and SD of the bone volume fraction (BV/TV), trabecular separation (TbSp), trabecular thickness (TbTh), trabecular number (TbN), trabecular connectivity density (ConnD), and bone mineral density (BMD) of the tuber coxae (TC) bone biopsies.

	**TC bone biopsies**				
**Days**	**BV/TV (%)**				
	**Con**	**T**_**0**_	**T**_**84**_	**T**_**0+84**_	**Average**
84	32.9 ± 4.7	33.9 ± 4.6	36.0 ± 9.4	35.1 ± 3.0	34.5 ± 5.8
165	33.8 ± 3.0	37.0 ± 4.4	39.1 ± 6.9	36.5 ± 2.7	36.6 ± 4.8
P-Value	0.80				0.04
**Days**	**TbSp (mm)**				
	**Con**	**T**_**0**_	**T**_**84**_	**T**_**0+84**_	**Average**
84	0.49 ± 0.08	0.48 ± 0.07	0.49 ± 0.11	0.47 ± 0.05	0.48 ± 0.07
165	0.54 ± 0.04	0.50 ± 0.05	0.49 ± 0.06	0.50 ± 0.04	0.51 ± 0.05
P-Value	0.44				0.02
**Days**	**TbTh (mm)**				
	**Con**	**T**_**0**_	**T**_**84**_	**T**_**0+84**_	**Average**
84	0.25 ± 0.08	0.25 ± 0.01	0.27 ± 0.02	0.25 ± 0.02	0.26 ± 0.02
165	0.27 ± 0.02	0.27 ± 0.01	0.29 ± 0.03	0.27 ± 0.02	0.28 ± 0.02
P-Value	0.99				< 0.001
**Days**	**TbN (mm** ^**-1**^ **)**				
	**Con**	**T**_**0**_	**T**_**84**_	**T**_**0+84**_	**Average**
84	1.37 ± 0.10	1.38 ± 0.14	1.34 ± 0.16	1.40 ± 0.13	1.37 ± 0.13
165	1.24 ± 0.07	1.29 ± 0.08	1.29 ± 0.09	1.29 ± 0.07	1.28 ± 0.08
P-Value	0.53				< 0.001
**Days**	**ConnD (mm** ^**-3**^ **)**				
	**Con**	**T**_**0**_	**T**_**84**_	**T**_**0+84**_	**Average**
84	10.0 ± 2.9	10.8 ± 3.9	10.7 ± 4.4	8.0 ± 3.8	9.8 ± 3.8
165	8.4 ± 3.2	9.8 ± 2.5	10.2 ± 3.5	9.6 ± 2.7	9.5 ± 3.0
P-Value	0.38				0.64
**Days**	**BMD (mg HA/cm** ^**3**^ **)**				
	**Con**	**T**_**0**_	**T**_**84**_	**T**_**0+84**_	**Average**
84	364 ± 77	390 ± 71	406 ± 68	365 ± 29	381 ± 64
165	363 ± 45	392 ± 76	396 ± 60	391 ± 43	386 ± 56
P-Value	0.78				0.67

Abbreviations: Con, control treatment no drugs administered; T_0_, group treated once on day 0 with clodronate disodium (CLO); T_84_, group treated once on day 84 with CLO; T_0+84_ group treated on day 0 and 84 with CLO.

No treatment differences were found for BV/TV, CtTh, TbSp, TbTh, TbN, and BMD of the LMC. Sex differences were found only for the BV/TV and BMD values, with greater values for females than males (p ≤ 0.006). No treatment, sex, or treatment-by-sex interaction differences were found for BV/TV, CtTh, TbSp, TbTh, TbN, and BMD of the L3 (Table 9).

**Table 9:** Treatment means and SD of the bone volume fraction (BV/TV), cortical thickness (CtTh), trabecular separation (TbSp), trabecular thickness (TbTh), trabecular number (TbN), and bone mineral density (BMD) of the left metacarpal medial condyle (LMC) and third lumbar vertebra (L3).

**Treatments**	**LMC**					
	**BV/TV** **(%)**	**CtTh** **(mm)**	**TbSp** **(mm)**	**TbTh** **(mm)**	**TbN** **(mm** ^**-1**^ **)**	**BMD** **(mg HA/cm** ^**3**^ **)**
Con	74.1 ± 4.6	1.34 ± 0.32	0.65 ± 0.17	0.47 ± 0.04	0.92 ± 0.16	3,770 ± 151
T_0_	74.9 ± 5.3	1.57 ± 0.46	0.64 ± 0.24	0.46 ± 0.05	0.94 ± 0.16	3,601 ± 227
T_84_	75.9 ± 3.1	1.55 ± 0.15	0.81 ± 0.30	0.47 ± 0.03	0.81 ± 0.30	3,786 ± 172
T_0+84_	76.6 ± 5.0	1.58 ± 0.24	0.60 ± 0.12	0.46 ± 0.05	0.95 ± 0.10	3,646 ± 265
P-value	0.55	0.33	0.17	0.95	0.18	0.16
**Sex**	**BV/TV** **(%)**	**CtTh** **(mm)**	**TbSp** **(mm)**	**TbTh** **(mm)**	**TbN** **(mm** ^**-1**^ **)**	**BMD** **(mg HA/cm** ^**3**^ **)**
Males	73.4 ± 4.2	1.47 ± 0.31	0.74 ± 0.24	0.46 ± 0.04	0.87 ± 0.16	3,596 ± 209
Females	77.4 ± 3.9	1.56 ± 0.32	0.61 ± 0.20	0.47 ± 0.04	0.94 ± 0.14	3,807 ± 170
P-value	0.006	0.33	0.09	0.25	0.13	0.003
**Treatments**	**L3**					
	**BV/TV** **(%)**	**CtTh** **(mm)**	**TbSp** **(mm)**	**TbTh** **(mm)**	**TbN** **(mm** ^**-1**^ **)**	**BMD** **(mg HA/cm** ^**3**^ **)**
Con	47.6 ± 3.7	0.94 ± 0.11	0.60 ± 0.04	0.32 ± 0.02	1.10 ± 0.05	1,718 ± 140
T_0_	49.2 ± 2.4	1.02 ± 0.16	0.56 ± 0.04	0.31 ± 0.01	1.15 ± 0.06	1,830± 148
T_84_	50.7 ± 4.0	1.06 ± 0.17	0.57 ± 0.05	0.32 ± 0.01	1.13 ± 0.06	1,844 ± 191
T_0+84_	47.8 ± 4.1	1.00 ± 0.13	0.58 ± 0.04	0.31 ± 0.02	1.12 ± 0.05	1,835 ± 139
P-value	0.21	0.35	0.26	0.64	0.22	0.21
**Sex**	**BV/TV** **(%)**	**CtTh** **(mm)**	**TbSp** **(mm)**	**TbTh** **(mm)**	**TbN** **(mm** ^**-1**^ **)**	**BMD** **(mg HA/cm** ^**3**^ **)**
Males	47.7 ± 4.1	0.97 ± 0.15	0.58 ± 0.04	0.31 ± 0.01	1.12 ± 0.05	1,789 ± 164
Females	50.0 ± 3.0	1.03 ± 0.14	0.58 ± 0.04	0.32 ± 0.02	1.12 ± 0.06	1,824 ± 156
P-value	0.06	0.20	0.65	0.30	0.93	0.57

Abbreviations: Con, control treatment no drugs administered; T_0_, group treated once on day 0 with clodronate disodium (CLO); T_84_, group treated once on day 84 with CLO; T_0+84_ group treated on day 0 and 84 with CLO.

### Serum bone biomarkers

Day 56 of PINP was excluded from the analysis due to laboratory error and lack of additional samples. No treatment or treatment-by-time interactions were found in SBB. However, time differences were observed for bone formation (BALP and PINP) and resorption markers (CTX-I and TRAP5b). Bone formation markers were higher on day 28 compared to day 0 (p ≤ 0.03). CTX-I was higher on days 0, 56, and 140 (p ≤ 0.03) compared to other days, while TRAP5b was higher on days 28 and 112 (p ≤ 0.02) compared to other days. RANKL had no changes over time (Table 10).

**Table 10:** Treatment means and SD of serum bone marker bone-specific alkaline phosphatase (BALP), procollagen type I amino-terminal propeptide (PINP), receptor activator of nuclear factor NF-ĸB ligand (RANKL), tartrate-resistant acid phosphatase isoenzyme 5b (TRAP5b), and carboxy-telopeptide of type I collagen cross-links (CTX-I).

	**BALP (ng/mL)**				
**Day**	**Con**	**T**_**0**_	**T**_**84**_	**T**_**0+84**_	**Average**
0	7.7 ± 5.0	6.3 ± 3.5	7.7 ± 3.8	6.0 ± 2.2	6.9 ± 3.7^c^
28	9.9 ± 8.1	9.4 ± 6.0	8.2 ± 4.5	9.3 ± 5.2	9.2 ± 5.9^ab^
56	12.0 ± 12.5	9.5 ± 6.6	9.3 ± 5.8	10.8 ± 6.8	10.4 ± 8.1^a^
84	9.0 ± 5.8	7.1 ± 4.0	7.1 ± 4.6	8.4 ± 3.9	7.9 ± 4.5^bc^
112	9.6 ± 9.9	8.6 ± 4.8	7.3 ± 3.7	8.0 ± 3.9	8.4 ± 6.0^abc^
140	10.3 ± 11.6	7.4 ± 3.7	6.9 ± 4.7	7.3 ± 3.1	8.0 ± 6.7^abc^
163	9.5 ± 10.0	6.2 ± 2.4	6.6 ± 4.5	6.1 ± 3.0	7.1 ± 5.8^bc^
P-Value	0.92				< 0.001
	**PINP (ng/mL)**				
**Day**	**Con**	**T**_**0**_	**T**_**84**_	**T**_**0+84**_	**Average**
0	1.7 ± 1.5	1.2 ± 0.9	1.8 ± 1.3	1.3 ± 0.7	1.5 ± 1.1^b^
28	2.1 ± 1.5	1.6 ± 1.3	1.8 ± 0.9	1.9 ± 1.4	1.8 ± 1.3^a^
56^1^	-	-	-	-	-
84	1.8 ± 1.2	1.4 ± 0.8	1.5 ± 0.9	1.8 ± 1.1	1.6 ± 1.0^ab^
112	1.5 ± 1.1	1.5 ± 1.0	1.4 ± 0.9	1.5 ± 0.8	1.5 ± 0.9^ab^
140	1.7 ± 1.3	1.4 ± 0.8	1.4 ± 0.9	1.7 ± 1.0	1.5 ± 1.0^ab^
163	1.4 ± 0.9	1.1 ± 0.5	1.2 ± 0.8	1.4 ± 0.8	1.3 ± 0.7^b^
P-Value	0.85				< 0.001
	**RANKL (ng/mL)**				
**Day**	**Con**	**T**_**0**_	**T**_**84**_	**T**_**0+84**_	**Average**
0	33.7 ± 14.3	37.6 ± 14.3	29.9 ± 9.3	37.8 ± 14.6	34.7 ± 13.2
28	23.6 ± 8.3	34.0 ± 18.8	29.4 ± 10.5	26.6 ± 7.0	28.0 ± 11.8
56	33.3 ± 7.4	28.0 ± 8.8	31.9 ± 6.9	31.5 ± 9.9	31.2 ± 8.2
84	27.1 ± 15.2	33.6 ± 14.7	22.2 ± 6.8	39.1 ± 14.7	31.0 ± 14.5
112	31.7 ± 16.7	24.5 ± 8.1	25.4 ± 13.5	35.6 ± 13.5	29.4 ± 13.7
140	24.0 ± 9.2	34.3 ± 17.3	30.4 ± 16.9	31.7 ± 13.7	30.0 ± 14.5
163	31.5 ± 17.5	36.2 ± 14.6	25.9 ± 15.8	24.5 ± 9.1	29.2 ± 14.5
P-Value	0.62				0.28
	**CTX-I (ng/mL)**				
**Day**	**Con**	**T**_**0**_	**T**_**84**_	**T**_**0+84**_	**Average**
0	10.3 ± 2.9	9.3 ± 3.3	9.4 ± 3.1	10.6 ± 3.7	9.9 ± 3.2^a^
28	8.0 ± 2.3	8.4 ± 2.5	7.5 ± 1.8	9.5 ± 2.8	8.3 ± 2.4^bcd^
56	9.7 ± 3.0	8.6 ± 1.6	9.2 ± 2.1	10.3 ± 3.0	9.5 ± 2.5^ab^
84	7.2 ± 1.5	8.2 ± 2.2	7.3 ± 1.7	9.4 ± 2.9	8.0 ± 2.2^cd^
112	7.1 ± 1.0	7.7 ± 2.2	7.2 ± 2.4	7.7 ± 1.1	7.4 ± 1.7^d^
140	9.0 ± 2.7	9.2 ± 1.9	8.2 ± 1.6	10.7 ± 3.0	9.3 ± 2.5^abc^
163	7.0 ± 2.2	8.5 ± 2.1	6.3 ± 1.7	9.1 ± 3.3	7.7 ± 2.6^d^
P-Value	0.63				< 0.001
	**TRAP5b (ng/mL)**				
**Day**	**Con**	**T**_**0**_	**T**_**84**_	**T**_**0+84**_	**Average**
0	22.7 ± 16.6	18.3 ± 9.3	22.2 ± 12.1	18.3 ± 8.1	20.4 ± 11.7^c^
28	34.9 ± 38.0	24.3 ± 15.9	26.6 ± 15.2	29.5 ± 15.7	28.8 ± 22.7^ab^
56	33.5 ± 39.8	21.2 ± 13.3	27.2 ± 22.8	28.4 ± 22.7	27.6 ± 25.8^bc^
84	33.2 ± 44.1	20.2 ± 10.4	21.5 ± 10.5	25.1 ± 11.3	25.0 ± 23.6^bc^
112	33.5 ± 29.1	29.7 ± 19.1	26.4 ± 16.7	32.0 ± 18.2	30.4 ± 20.7^a^
140	28.2 ± 29.0	20.9 ± 10.2	19.8 ± 10.1	22.3 ± 7.7	22.8 ± 16.4^bc^
163	26.7 ± 33.4	18.6 ± 9.2	18.0 ± 9.3	18.8 ± 5.9	20.6 ± 18.0^c^
P-Value	0.98				< 0.001

Abbreviations: Con, control treatment no drugs administered; T_0_, group treated once on day 0 with clodronate disodium (CLO); T_84_, group treated once on day 84 with CLO; T_0+84_ group treated on day 0 and 84 with CLO. Means followed by a common letter are not significantly different by the Tukey-Kramer test at the 5% level of significance.

^1^Day 56 PINP data excluded from the analysis due to laboratory error and lack of sufficient samples.

No treatment differences were found for BALP/CTX-I and CTX-I/TRAP5b indexes. However, time differences were observed. BALP/CTX-I on days 28 and 56 were higher (p ≤ 0.009) than day 0. In contrast, CTX-I/TRAP5b on days 28, 84, and 112 were lower (p ≤ 0.02) than on days 0 and 56 (Table 11).

**Table 11:** Treatment means and SD of the remodeling ratio (BALP/CTX-I index) and resorption ratio (CTX-I/TRAP5b index).

	**BALP/CTX-I index**				
**Day**	**Con**	**T**_**0**_	**T**_**84**_	**T**_**0+84**_	**Average**
0	0.81 ± 0.56	0.80 ± 0.56	0.93 ± 0.60	0.62 ± 0.29	0.79 ± 0.51^b^
28	1.43 ± 1.28	1.31 ± 0.90	1.19 ± 0.53	1.17 ± 0.73	1.28 ± 0.83^a^
56	1.41 ± 1.64	1.07 ± 0.69	1.16 ± 1.02	1.13 ± 0.69	1.19 ± 1.05^a^
84	1.25 ± 0.77	0.94 ± 0.63	0.99 ± 0.61	1.00 ± 0.59	1.05 ± 0.64^ab^
112	1.35 ± 1.26	1.12 ± 0.57	1.16 ± 0.79	1.09 ± 0.62	1.18 ± 0.83^a^
140	1.25 ± 1.49	0.83 ± 0.44	0.88 ± 0.61	0.76 ± 0.41	0.93 ± 0.85^ab^
163	1.35 ± 1.27	0.75 ± 0.26	1.12 ± 0.77	0.77 ± 0.46	1.00 ± 0.81^ab^
P-Value	0.99				< 0.001
	**CTX-I/TRAP5b index**				
**Day**	**Con**	**T**_**0**_	**T**_**84**_	**T**_**0+84**_	**Average**
0	0.67 ± 0.47	0.67 ± 0.46	0.52 ± 0.27	0.67 ± 0.37	0.64 ± 0.39^a^
28	0.36 ± 0.19	0.49 ± 0.40	0.32 ± 0.15	0.38 ± 0.22	0.39 ± 0.25^bc^
56	0.63 ± 0.53	0.54 ± 0.26	0.52 ± 0.29	0.61 ± 0.57	0.58 ± 0.42^a^
84	0.42 ± 0.25	0.51 ± 0.28	0.39 ± 0.15	0.46 ± 0.28	0.45 ± 0.24^bc^
112	0.33 ± 0.21	0.34 ± 0.18	0.36 ± 0.22	0.33 ± 0.19	0.34 ± 0.19^c^
140	0.56 ± 0.40	0.54 ± 0.28	0.50 ± 0.22	0.57 ± 0.33	0.54 ± 0.30^ab^
163	0.46 ± 0.27	0.53 ± 0.21	0.43 ± 0.20	0.55 ± 0.34	0.49 ± 0.26^ab^
P-Value	0.84				< 0.001

Abbreviations: Con, control treatment no drugs administered; T_0_, group treated once on day 0 with clodronate disodium (CLO); T_84_, group treated once on day 84 with CLO; T_0+84_ group treated on day 0 and 84 with CLO. Means followed by a common letter are not significantly different by the Tukey-Kramer test at the 5% level of significance.

### Sulfated glycosaminoglycans (GAG)

**Fig 2:**
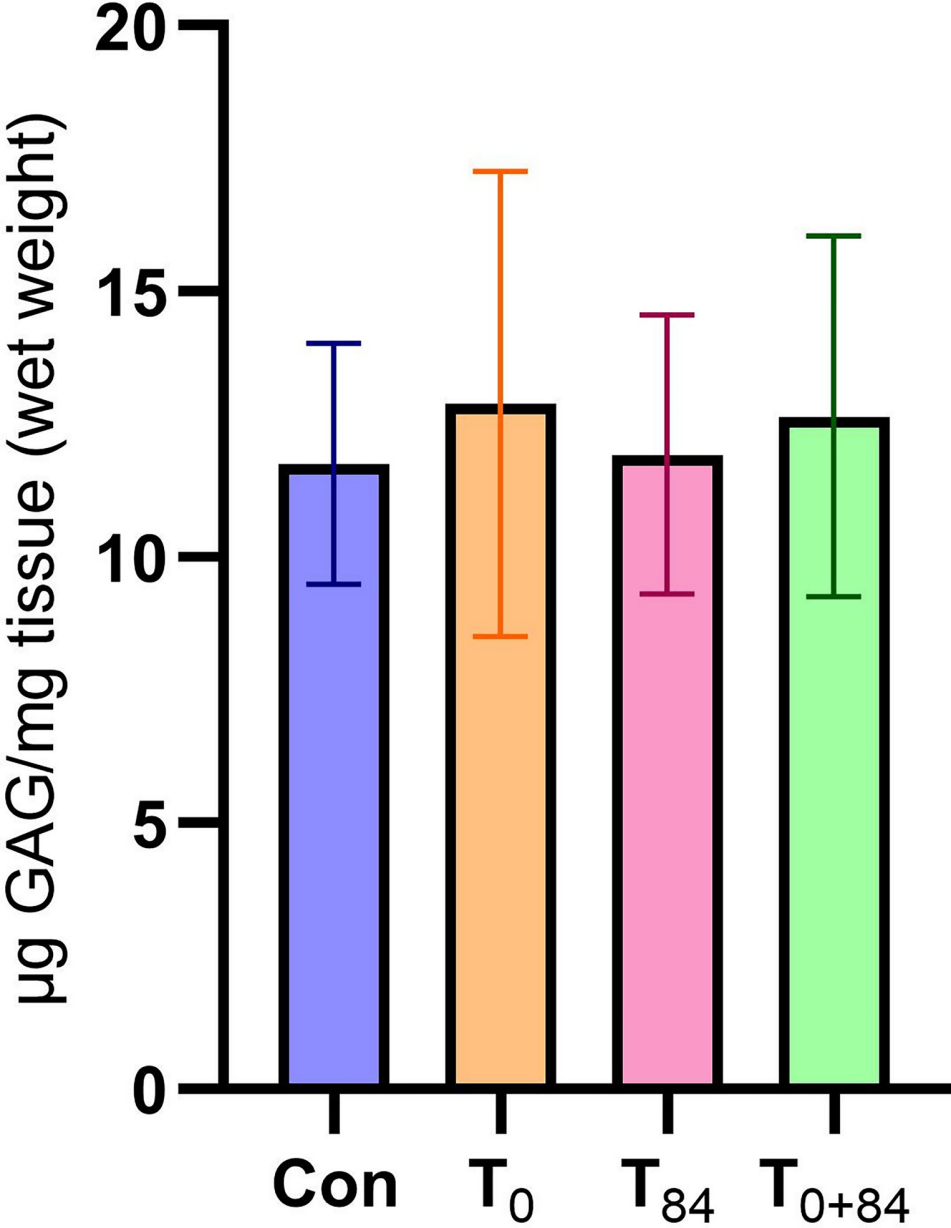
Sulfated glycosaminoglycan (GAG) content from the right proximal radius cartilage post-papain digestion (μg GAG/mg tissue). No differences between treatments were found (p = 0.82). Abbreviations: Con, control treatment no drugs administered; T_0_, group treated once on day 0 with clodronate disodium (CLO); T_84_, group treated once on day 84 with CLO; T_0+84_ group treated on day 0 and 84 with CLO.

Cartilage GAG content did not differ between treatments (Fig 2).

## Discussion

Bisphosphonates are one of the most common drugs used to treat and prevent bone resorption via osteoclast impairment [1, 3, 4, 75]. However, the effects of bisphosphonates on immature skeletal development while undergoing exercise are largely unknown [24, 25, 30, 32, 33]. Therefore, we sought to determine the effects of a single or repeated i.m. administration of CLO in a juvenile sheep model subjected to exercise. We failed to reject our null hypotheses, as no differences were detected among treatments. However, sex differences were realized for multiple outcome measures including BW, height, right MC_3+4_ (dimensions), L4 (dimensions and BT), and LMC (BV/TV and BMD). Also, BW, height, TC biopsies (BV/TV, TbSp, and TbTh), and SBB (BALP, PINP, CTX-I, and TRAP5b) outcome measures were noted to change over the study period.

Treatments with bisphosphonates impair osteoclast function [1, 3]; these biological changes can be detected through SBB [6, 7], changes in BMD [6, 7], and/or BT [8, 9]. Our results suggested that CLO did not produce any measurable effects on bone metabolism in juvenile sheep subjected to exercise. Our findings are similar to previous reports that showed bisphosphonates administered to large animals (i.e., horses) were clinically effective in reducing musculoskeletal pain [20, 47, 76, 77], without causing changes in SBB, BMD, micro-CT measurements, and/or BT [19, 20, 46, 47, 76, 77] using FDA-approved doses [46, 78]. This may be due to the lower dose used in large animals compared to humans. We used a dose in sheep that resembled the pharmacokinetic values of therapeutic CLO administered in horses [48]. The dose used in horses (1.8 mg/kg) has shown clinical efficacy in reducing musculoskeletal pain in multiple studies [20, 46, 47, 77]. However, humans treated for osteoporosis receive 200 mg i.m. every 2 weeks (approximately 2.7 mg/kg based on a 75 kg person) which results in detectable BMD increases [79]. A linear relationship is described between the dose and frequency of bisphosphonates and their effects [8, 79, 80], where a greater dose correlates with a greater inhibition of osteoclasts and subsequent effects on bone turnover. Therefore, lower CLO doses may lead to clinical effects without significant antiresorptive outcomes in the skeleton [81]. This study did not assess pain or pain control related to CLO administration. However, the results suggest that future studies could investigate low dose CLO therapy as a palliative treatment with minimal effects on BMD or growth in juveniles.

Clodronate has a lower anti-resorptive potency compared to other bisphosphonates which may explain the lack of measurable bone effects. Bisphosphonates are categorized into nitrogen-containing and non-nitrogen-containing bisphosphonates [82, 83], and it is well established that the non-nitrogen-containing bisphosphonates (e.g., CLO) have a lower binding affinity and antiresorptive potency than nitrogen-containing bisphosphonates (e.g., ibandronate or zoledronate) [82, 83]. Consequently, the class of bisphosphonate, coupled with the lower dose employed in this study, may have fallen below the threshold needed to produce detectable osteoclast inhibition. Notably, exercise and age influence the bisphosphonate distribution in the body. Exercise decreases the glomerular filtration rate [84], which in turn reduces the excretion of CLO and enhances blood supply to the bones [85], potentially increasing CLO availability within the skeletal system. Additionally, age influences bisphosphonate absorption by the bones, with higher absorption expected in young populations due to greater bone turnover compared to adults [86, 87]. Even though this study used juvenile animals subjected to exercise, which could have increased their exposure to CLO, no measurable skeletal effects were observed. Administering a higher dose and/or evaluating a higher-intensity training may result in detectable changes in bone parameters.

Although SBB and TC biopsies did not show any time-by-treatment differences, there was an effect of time. In this study, bone formation markers increased on days 28 (BALP and PINP) and 56 (BALP), while CTX-I, which is a marker of osteoclast activity [71], decreased on several days. Similarly, the BALP/CTX-I remodeling index increased on days 28 and 56, suggesting further increase in bone formation on those days. This idea is supported by an increase observed on BV/TV driven by an increase of TbTh in the TC biopsies. However, TRAP5b, a marker of osteoclast numbers [71, 72], increased on days 28 and 112, while RANKL did not increase during the study, indicating that there was not a significant osteoclast activation [88]. TRAP5b can be released from both mature and immature osteoclasts [72, 89]; hence, an increase in this marker may not necessarily indicate increases in bone resorption. Moreover, when CTX-I is evaluated in relation to TRAP5b (CTX-I/TRAP5b), it may provide a more comprehensive understanding of the biological activity of bone resorption. The CTX-I/TRAP5b resorption index showed a consistent decrease on days 28, 84, and 112, in similar fashion to the response in BALP, PINP, and CTX-I. Future studies should include other markers, such as cathepsin K, which is released by mature osteoclasts only [72].

The observed changes in TC biopsies could be explained by the exercise protocol, as physical activity favors increased bone formation and decreased bone resorption, as observed in the SBB [90]. However, TC is a non-weight bearing bone and may not respond to exercise [91]. Therefore, increases in BALP and PINP (bone formation markers), and no changes in RANKL with decreases in CTX-I (bone resorption markers), may be more related to animal growth than exercise. Sheep were approximately 80% developed at the beginning of the study and showed significant growth during the study period. Increases in BV/TV and TbTh are commonly found in the iliac bone of growing children [92], which is similar to the TC changes observed in the juvenile sheep. For this reason, future studies should include a sedentary control group in order to differentiate between the effects of exercise and/or growth when using juvenile animals.

Sex differences were observed in several parameters, including BW, height, right MC_3+4_ and L4 dimensions, L4 BT, and LMC BV/TV and BMD. Sexual dimorphism is a natural characteristic of this species, particularly regarding body size [93, 94]. This study utilized castrated males. Orchiectomy leads to a decrease in sex hormones, resulting in reduced bone mass due to decreased bone accrual during growth and increased bone resorption [95–97]. Therefore, the reductions in LMC BV/TV and BMD of males compared to females can be explained by the reduction of sex hormones. Furthermore, the castrated male L4 specimens exhibited decreased compressive stress at failure and an increased modulus of elasticity compared to the intact female L4 specimens. This apparent contradiction between decreased compressive stress at failure and increased modulus of elasticity can be explained by the lack of circulating sex hormones as well. Studies show that vertebrae compensate for bone loss from reduced sex hormones by cranio-caudally rearranging their collagen and HAP [98, 99]. This rearrangement results in an increased modulus of elasticity, despite a decrease in compressive stress at failure [98, 99]. Although L4 specimens did not undergo micro-CT analysis, L3 microstructure was analyzed and no differences between sexes were found. However, there was a trend (p = 0.06) towards increased BV/TV in females compared to males in L3 specimens, suggesting that even small changes in BV/TV may significantly affect the mechanical structure of the lumbar vertebrae. Thus, castrated male L4 specimens may have experienced cranio-caudal collagen and HAP rearrangement as a compensatory response, although this was not addressed in the present study. Notably, no significant BMT sex differences in weight-bearing bones were found (e.g., right MC_3+4_), indicating that the lack of sex hormones’ effect on weight-bearing bones can be compensated for by physical activity [97, 100–102]. Based on our findings, future studies should consider the effects of castration influencing sex hormone levels and bone microstructure. This will aid in translating results from the sheep model to other species, including humans.

In addition to the PE, lameness evaluations showed differences in the T_0_ group only, with an increase in the probability of lameness observed on day 94. This increase is likely attributed to a temporary increase in lameness following the bone biopsy procedure performed on day 84. In particular, one individual remained consistently lame following TC biopsy; this individual was eventually removed from the future analysis. No other increases in probability of lameness were detected, suggesting that the sheep tolerated the exercise protocol well and CLO did not result in significant lameness.

Detectable levels of CLO are achieved in synovial fluid following i.m. administration in horses [103]. To assess the potential effect of CLO on cartilage health in juvenile, exercising animals, GAG content was measured from cartilage samples. GAG analysis of cartilage samples from the proximal surface of the radius did not reveal treatment differences. This finding is consistent with a previous *in vitro* investigation that evaluated the impact of various concentrations of CLO on cartilage explants, chondrocytes, and synoviocytes and found no evidence of either cytoprotective or cytotoxic effects [104]. High doses of bisphosphonates have shown *in vitro* cytotoxicity in joint tissues, such as bovine chondrocytes [105] and equine cartilage [106]. Bisphosphonate content in the synovial fluid of joints was not measured in the current study. Future experimental studies should explore different doses of CLO *in vivo*, as low doses of CLO may provide a clinical effect without cartilage toxicity.

Another known effect of bisphosphonates is their analgesic properties. CLO has also been used to treat chronic pain in humans [107, 108]. Analgesic effects of CLO may be attributed to blockade of the vesicular nucleotide transporter [109]; CLO is the strongest bisphosphonate inhibitor of this transporter [110]. These analgesic effects are desirable in painful bone-related conditions, such as osteogenesis imperfecta or bone cancer [107]. Bisphosphonates’ analgesia can have long-lasting effects [107], and can be independent of antiresorptive effects [81], as demonstrated in multiple studies where analgesic effects are found in the absence of skeletal changes [19, 20, 46, 77, 78]. Therefore, low doses of CLO may be used as treatment for refractory pain without skeletal changes [81]. The analgesic effects of CLO were not evaluated in this juvenile sheep model. However, future studies using this model are warranted to assess the analgesic effects of low doses of CLO considering the lack of negative skeletal effects in growing, active individuals. This could be particularly promising for children experiencing musculoskeletal pain for which long-term pain management with non-steroidal anti-inflammatories, opioids or steroids could result in significant morbidity.

While we were able to compare treated animals to an untreated control group in the exercising study population, without a sedentary group we are not able to identify the effect of exercise, exercise intensity, or growth. In addition, SBB can show changes within hours after exercise. Having more sample points may have allowed for a better characterization of the SBB’ response over time [90]. Further, bisphosphonates may produce glomerulosclerosis [111], and additional blood and urine samples may have helped to characterize the risk of CLO use by exercising individuals. Finally, the sex should be considered in future studies as castration may have influenced some skeletal outcomes.

## Conclusions

In conclusion, our study found no measurable skeletal effects on juvenile, exercising sheep following the administration of a single or repeated dose of 0.6 mg/kg of CLO. The lack of effects could be attributed to the lower dose used in animals. Further investigation is required to explore bisphosphonates’ analgesic effects, as low doses of CLO may provide analgesic benefits with minimal negative skeletal effects. Long-lasting analgesia without negative skeletal effects may be particularly advantageous considering the morbidity associated with long term use of other commonly used analgesics such as non-steroidal anti-inflammatories, steroids, and opioids. Future studies should explore effects of sex, include a sedentary group, and investigate additional doses in conjunction with exercise. Moreover, additional studies should investigate the effects of more potent bisphosphonates, such as zoledronic acid, or newer bisphosphonates, such as lidadronate (IG9402) [2], on the skeleton of juvenile, active individuals.

## Supporting information

S1 Fig Diagram and photographs of facilities used for the exercise protocol. **A**: satellite image is used to show the location of indoor pens (dark blue, 21.6 m^2^ each), the distance between the walker and indoor pens (light blue, 32 m of fencing), the 20 m diameter exerciser (Q-Line Horse Exerciser, Aromas, CA, USA). **B**: Panoramic view of the high-speed walker. **C**: Inside view of sheep walking at 1.3 m/s. Sheep walked clockwise and counterclockwise on alternate days.
(TIFF)

S2 Fig Placement and diagram of a right MC_III&IV_ subjected to 4-point bending on an Instron. **A**: Placement of the right fused third and fourth metacarpal (MC_III&IV_) for four-point bending test using electromechanical testing system (60 kN load cell, MTSCriterion, Model 43, Eden Prairie, MN). Specimens were kept with wrapping paper to avoid dehydration and loss of fragments after fracture failure. **B**: Diagram The load exerted (*F*) on the bone is depicted as the Instron measures the bone’s displacement (*V*). *L* is the span length, which was 51.9 mm; *a* is the distance between *F* and the supports on either end of the bone with a value of 3.8 mm. Each support was 24.5 mm wide. Adapted from Logan et al. [48].
(TIFF)

S3 Fig Preparation of vertebral bodies prior to embedding with polyurethane resin. Dissection from soft tissues, transverse processes, and dorsal arch.
(TIFF)

S4 Fig 3D holder and molds used previous compressive test of lumbar spine. **A**: 3D printed holder to keep cranial and caudal-leveled surfaces. **B**: Silicone molds and 3D print used holder for polyurethane resin embedding (TC-808, BJB Enterprises, Tustin, CA, USA) of vertebral bodies prior to compression tests.
(TIFF)

S5 Fig Lateral view of fourth lumbar vertebra for electromechanical testing compressive test. The compressive test was performed using a 100kN load cell (Instron model 5982 Norwood, MA, USA) and a flat 3 mm thick metal plate.
(TIFF)
