# Supplementary figures and images for: Clodronate disodium does not produce measurable effects on bone metabolism in an exercising, juvenile, large animal model
Source: PLoS One. 2024 Apr 16;19(4):e0300360. doi: 10.1371/journal.pone.0300360 (PMC11020481; doi:10.1371/journal.pone.0300360)

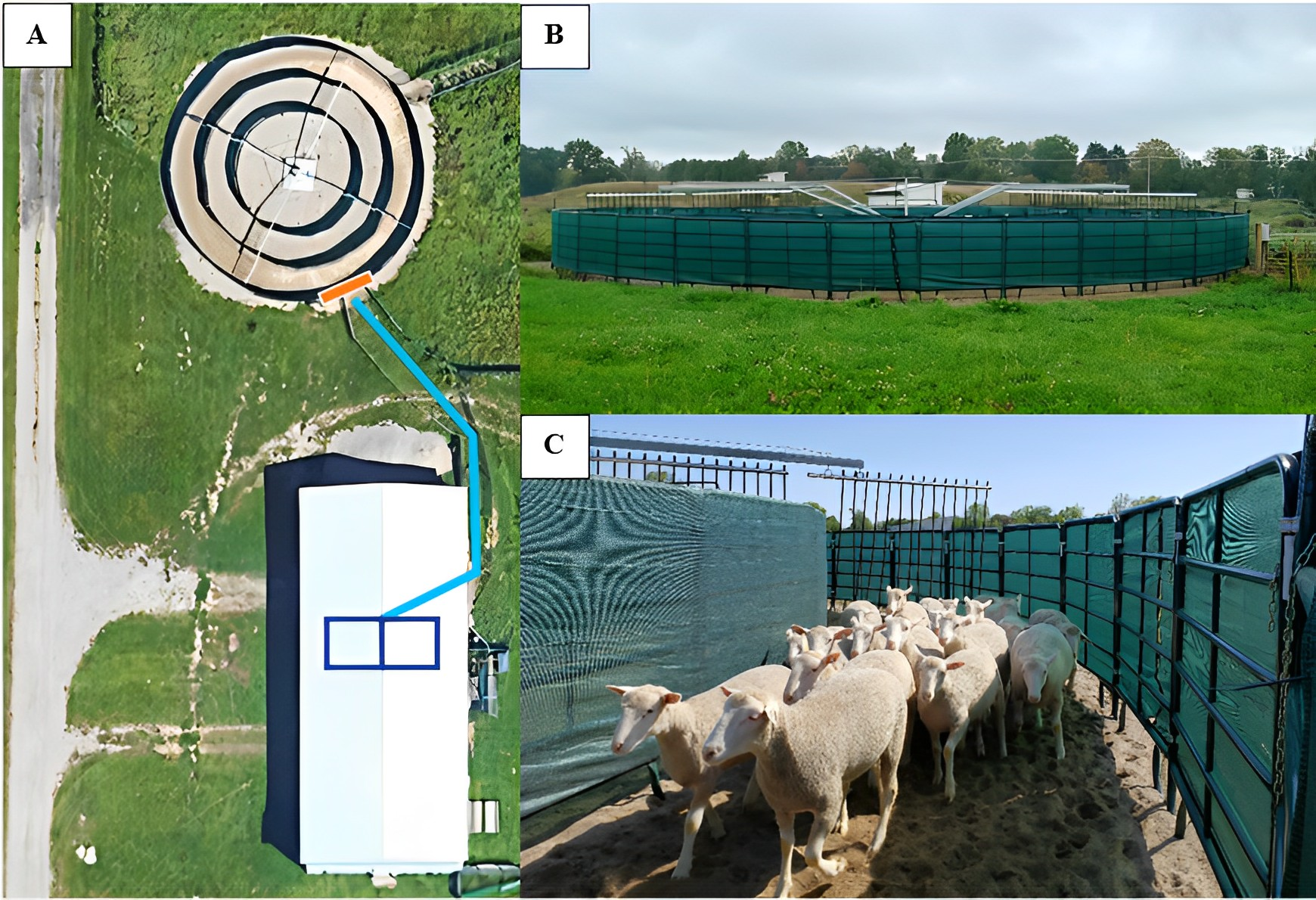

Supplement: S1 Fig — A: satellite image is used to show the location of indoor pens (dark blue, 21.6 m2 each), the distance between the walker and indoor pens (light blue, 32 m of fencing), the 20 m diameter exerciser (Q-Line Horse Exerciser, Aromas, CA, USA). B: Panoramic view of the high-speed walker. C: Inside view of sheep walking at 1.3 m/s. Sheep walked clockwise and counterclockwise on alternate days. (TIFF) [file pone.0300360.s001.tiff]

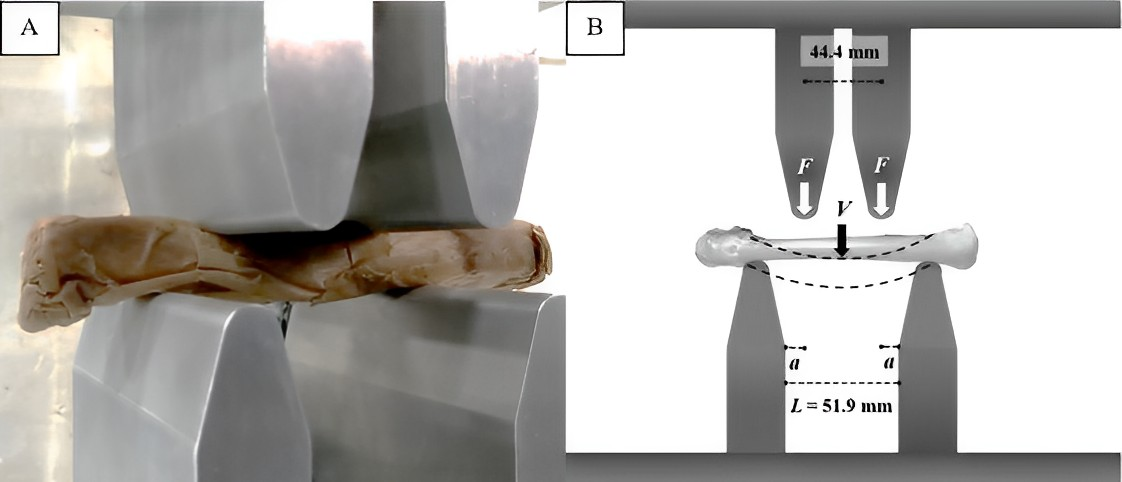

Supplement: S2 Fig — A: Placement of the right fused third and fourth metacarpal (MCIII&IV) for four-point bending test using electromechanical testing system (60 kN load cell, MTSCriterion, Model 43, Eden Prairie, MN). Specimens were kept with wrapping paper to avoid dehydration and loss of fragments after fracture failure. B: Diagram The load exerted (F) on the bone is depicted as the Instron measures the bone’s displacement (V). L is the span length, which was 51.9 mm; a is the distance between F and the supports on either end of the bone with a value of 3.8 mm. Each support was 24.5 mm wide. Adapted from Logan et al. [48]. (TIFF) [file pone.0300360.s002.tiff]

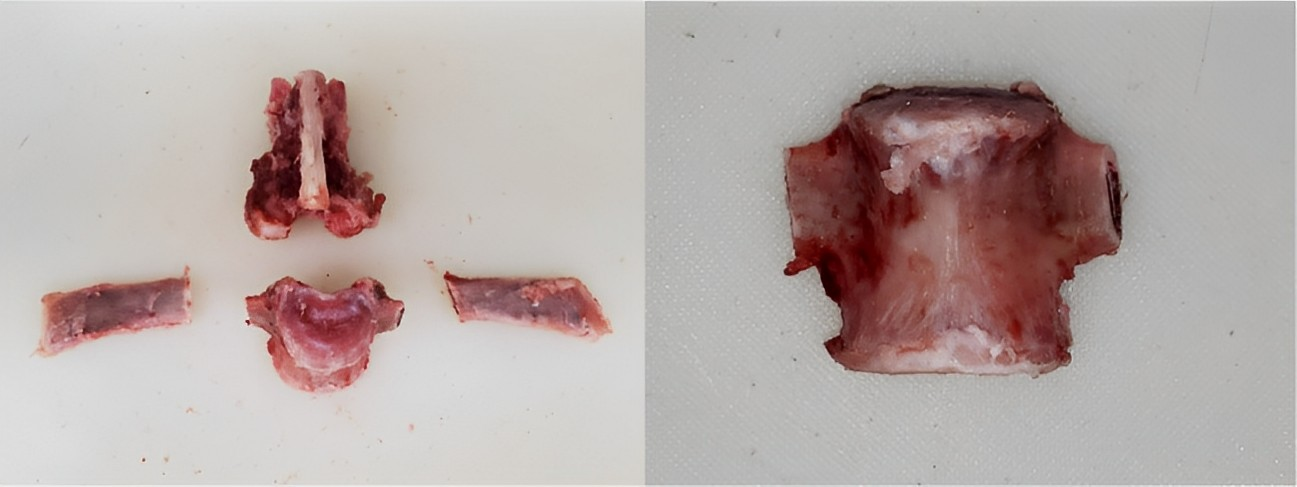

Supplement: S3 Fig — Dissection from soft tissues, transverse processes, and dorsal arch. (TIFF) [file pone.0300360.s003.tiff]

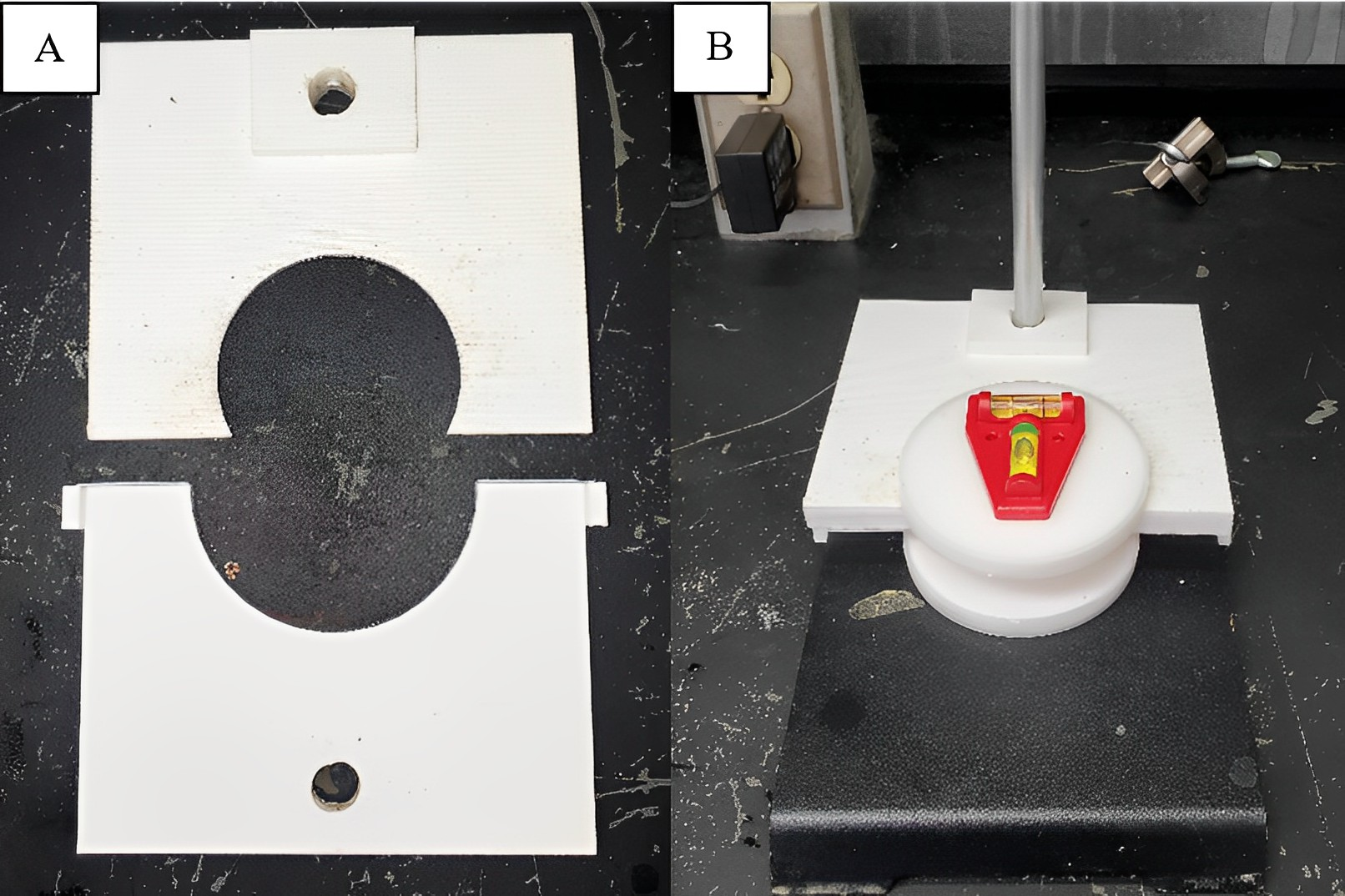

Supplement: S4 Fig — A: 3D printed holder to keep cranial and caudal-leveled surfaces. B: Silicone molds and 3D print used holder for polyurethane resin embedding (TC-808, BJB Enterprises, Tustin, CA, USA) of vertebral bodies prior to compression tests. (TIFF) [file pone.0300360.s004.tiff]

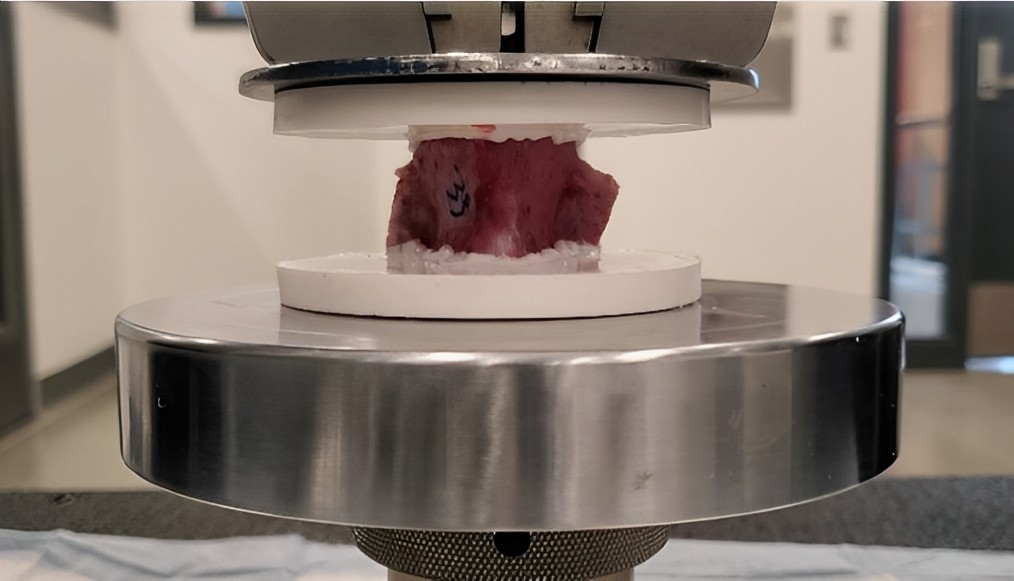

Supplement: S5 Fig — The compressive test was performed using a 100kN load cell (Instron model 5982 Norwood, MA, USA) and a flat 3 mm thick metal plate. (TIFF) [file pone.0300360.s005.tiff]
